# New York Academy of Medicine—Section in Orthopaedic Surgery—Meeting of February 17, 1899

**Published:** 1899-04

**Authors:** 


### New York Academy of Medicine—Section in Orthopaedic Surgery—Meeting of February 17, 1899.

### HYPERTROPHY OF THE TIBIA.

Dr. S. Ketch presented a girl 4 years of age whose right tibia was greatly lengthened and thickened with decided anterior bowing. He had first seen the patient in December, 1898. The epiphyses were thickened, but the enlargement was not confined to them. It was most marked at the middle of the shaft, but included the whole bone, as was seen by the X-rays. Length: right leg, 19½; left leg,

$18\frac{5}{8}$; right tibia, $9\frac{1}{4}$; left tibia, $8\frac{3}{4}$. Circumference: right thigh, $9\frac{1}{2}$; left thigh, $10\frac{1}{4}$; right calf, $8\frac{5}{8}$; left calf, $7\frac{7}{8}$. The disease had begun eighteen months ago, with a small lump on the leg and pain at night and when she walked. This was Dr. Ketch's second patient of the kind. The first one was a girl 11 years of age, who had been presented to the section in November, 1897, had been operated on for the purpose of shortening and straightening the bone, and had again been before the section in March, 1898, with resulting improvement and ability to walk about. [See TEXAS MEDICAL JOURNAL, August, 1898, pp. 64, 65.—ED.]

The bone had been found to be solid, the cavity being obliterated. Neither of the patients had received any benefit from anti-syphilitic treatment. There was doubt as to the cause of this growth of the bone. It was not improbable that the trouble began in the periosteum. It was a question whether something ought not to be done early in the way of an operation to arrest the process, such as an incision through the periosteum which might at least relieve the tension.

Dr. T. H. Myers said that this affection was extremely rare. He did not think that any drug could produce a material improvement, thought it might prevent further progress of the disease. Such cases were sometimes assumed to be syphilitic for lack of better information, though no history or symptoms of that infection could be elicited.

Dr. V. P. Gibney suggested a linear incision through the periosteum, and if that could be done with perfect safety going further by denuding the bone from the anterior surface and shaving off the redundant portion, suturing the periosteum and letting it heal primarily. The growth in length could not be stopped except by attacking the epiphysis, which would be hazardous.

Dr. H. Gibney said that in addition to the treatment which had been suggested, he would go further and complete the operation, straightening the leg by the removal of a wedge-shaped piece of the bone and maintaining the correct position by plaster of Paris dressings.

Dr. Myers thought that incision would only relieve the pain. He would not operate until the child had attained its growth or the disease had stopped.

Dr. G. R. Elliot said that it was of pathological interest that the tibia alone was affected while the fibula remained normal. There was but little deformity compared with the decided bowing which had been an indication for operation in Dr. Ketch's former patient, in whom the pathological findings were diffusely distributed throughout the entire thickness of the bone. He asked what effect tying the nutrient artery of the bone would have on the progressive atrophy.

Dr. Ketch said it would probably stop the growth of the bone. bone.

Dr. Elliott suggested the possibility of resulting necrosis.

Dr. A. B. Judson said that if the whole limb were affected symmetry might possibly be promoted during the growing period by checking the vascular supply of the larger limb, by bandaging or lacing the whole limb, and increasing the vascular supply of the smaller limb by venous compression. At the same time the functional activity of the one could be lessened and that of the other increased by use of an ischiatic crutch or other apparatus having the same effect, with a high sole under the shorter limb. But as the diagnosis was absent and the pathology unsettled, he could not suggest a reasonable treatment.

Dr. Ketch said that at an earlier stage some of the operative procedures suggested might have arrested or prevented the abnormal growth of the bone, but, on the other hand, they might have promoted it. He was opposed to the removal of a portion of the bone during the growing period. As the parents of the child desired active treatment, an incision might be recommended as likely to stop the pain, which he thought was due to tension.

### ENLARGEMENT OF EPIPHYSES.

Dr. Myers presented a girl 16 months of age whom he had seen for the first time on January 10, 1899. The epiphyses of the radii, femora, tibiæ and the entire phalanges of several fingers were enlarged. The joints of the ankles, knees, fingers, wrists and the right elbow were swollen and somewhat restricted in their motions. The enlargement at the ankle joint was peculiar, several of the tarsal bones sharing in it. She walked with difficulty, with knees and hips flexed. Flexion of the knees and unwillingness to walk had been observed immediately after an attack of cholera morbus in October, 1898. The knees were kept a little flexed, and there was a very slight effusion in these joints. The child did not sleep well, but otherwise seemed to be in good health. Potassium iodide, gr. iv-viii, had been given t. i. d. for a month without improvement. The teeth were not notched. There was no syphilitic hystory. It was not typical scurvy. The child had been for three months on a general diet including eggs, meat, potatoes and fruit. It was certainly not a typical case of rickets. She had cut teeth early and walked at ten months, the head was well formed and the abdomen not prominent. The diagnosis remained uncertain.

Dr. Ketch said that the obvious feature of the case was a very exaggerated change in nutrition—an overgrowth of some kind, the effect of some not so obvious diathetic cause. He had seen localized changes in scorbutus which were very similar.

Dr. V. P. Gibney said that the changes were similar to those seen in chronic rheumatoid arthritis, which he had repeatedly seen in typical forms in children seven and eight years of age, and he did not see why it should not attack a child sixteen months old. This, however, would not explain the growth of the long bones and phalanges. His first thought was of scorbutus, but the condition would have disappeared with the child on the diet stated. Syphilis could be excluded. If pushed for an opinion, he would say it was a case of multiple bone tuberculosis, a condition which would be less easily excluded than any of the others mentioned. The boggy feeling of the joints, the fact that there was effusion in the joints and the statement that flexion of the knees and an unwillingness to walk had followed an attack of cholera infantum, all supported the view that it was an instance of bone tuberculosis. He would raise the question whether synovitis was not one of the earliest signs of tuberculosis in a child. He advised putting the child in a wire cuirass and keeping the limb extended. It was not good to allow the child to walk.

Dr. Ketch said that primary synovial tuberculosis was rare in children.

Dr. Judson had noticed the contraction of the knees and hips, but thought it was not the result of the reflex muscular action of joint disease, and that the fact that the contractions were nearly symmetrical pointed to a more general cause than tuberculosis of the joints affected. He did not think that synovitis was an early incident of osteitis and that primary synovitis could be differentiated by the absence of the usual signs of osteitis, which were muscular atrophy and reflex action and a prolonged history of inconstant lameness and pain. Synovitis should not be considered as liable to run into osteitis, although practically it was well to relieve a synovitic joint from weight bearing.

Dr. Ketch said that he had rarely seen synovitis as an early sign of tuberculosis.

Dr. V. P. Gibney said that the focus of diseased bone might suffer a traumatism and thus cause an extension of the process and give rise to this outward manifestation. He recalled a case seen twenty years ago. The child's knee was full of fluid. It was thought surely to be synovitis, and a glowing prognosis of recovery in a few weeks was made, but after six or seven years' treatment recovery took place with a stiff knee. Primary osteitis with secondary synovial distension occurred before the gross signs of the osteitis which called the attention of the practitioner to some trouble in the knee. At this stage the trouble could be cured.

Dr. Elliott said that fluid in a joint immediately after a traumatism pointed clearly to a synovitis directly due to traumatism. If tuberculosis followed, it resulted from a further injury to the bone itself, which made a proper nidus for the tubercular growth. In other words, a dual injury and the fluid in the joint was entirely distinct from the true tubercular lesion, and in no way connected with it. The later tubercular development might delay the absorption of the primary synovial excess, and thus the latter might come to complicate the tubercular joint.

Dr. Myers had seen effusion early in tubercular joint disease, but did not consider it of diagnostic value. In spite of the fact that the patient had had apparently an anti-scorbutic and anti-rachitic diet, he could not help thinking that the trouble was due to one of these diseases rather than to tuberculosis. The child was not very sick. The principal changes were in the epiphyses and phalanges, and seemed to him to be due to some form of nutritional disease. The congested epiphyses could fully account for the pain and tenderness, but he would adopt the suggestion made and protect the joints by keeping the child quiet.

### CASES OF COXA VARA.

Dr. Myers also presented a boy 8 years of age who had waddled, and was walking worse every year, since he began to walk. His muscles were strong. A certain rigidity of all the muscles of the lower extremities made examination somewhat difficult. The motions of the hip joints, especially flexion and abduction, were somewhat limited. There was no dislocation, but the neck of the femur was seen in the skiagram to be bent down as in coxa vara. The diet had been very good. The boy was a little bow-legged and flatfooted.

Dr. H. Gibney found no shortening and trochanters but slightly above the line. He thought the waddling might be due to flat feet.

Dr. V. P. Gibney said that the radiograph showed forward rotation and a little bending backwards of the femoral neck at its junction with the shaft.

The opinion was expressed by several speakers that the boy had coxa vara in a mild and not strictly typical form.

Dr. Elliott thought that the condition dated from early rachitis in all probability. The picture was a logical one, and the femoral neck had changed simultaneously with the bowing of the legs, both having been more or less plastic.

Dr. Ketch said that the traces of rachitis were obvious. Coxa vara was sometimes made to include cases that were not dependent on bending of the bone. Some cases were due to deviations caused by abnormal epiphyseal growth resulting in a change in the angle of the neck of the femur. On the other hand, the peculiar gait of coxa vara was not infrequently attributed to knock-knees or bow-legs.

Dr. Judson said that coxa vara might be considered to mean an abnormal or varous relation of the neck of the shaft caused by lesions of different kinds, all of which were not yet recognized.

Dr. V. P. Gibney said that in coxa vara we had found one new disease or condition to rule out in our study of hip disease. Many cases of hip disease in adolescents which recover and have relapses, but never get very bad, having from one-half to three-fourths inch shortening, were really cases of coxa vara.

Dr. Ketch presented a boy aged eleven years, who had had a limp (left leg) in winter but not in summer, for three years. Pain and inability to walk on rising disappeared entirely in the afternoon. There had been no history of rickets or rheumatism. Abduction was limited, especially in flexion. Outward rotation abnormally free, trochanter one-half inch above the line, no atrophy. R., 28; L., 27 5-8. The skiagraph showed a change in the angle of the neck.

TREATMENT OF COXA VARA.

Dr. Judson suggested mechanical means for permitting locomotion while the affected part is relieved from the weight of the body as long as the bone was in a growing or plastic state.

Dr. V. P. Gibney said that when the affection was single good results could be obtained from the use of the hip-splint. He saw no objection to the wearing of a jointed splint for some months, affording, not absolute but modified, protection, enough to shut out traumatism.

Dr. H. Gibney said that the ischiatic crutch for this purpose was easily adjusted and comfortably worn and allowed the limb to hang free.

Dr. Myers said that when both femora were affected mechanical protection was attended with difficulties, and it was not easy to keep the adolescent patient, like the one he had presented, quiet.

Dr. Judson suggested the use of a bicycle.

Dr. Ketch in such a case would improve the general nutrition and prepare the parents for a long wait.

### PAIN RELIEVED BY TRACTION.

Dr. Myers related the history of a patient 26 years of age, who had suffered five and one-half years from rheumatism in the ankles, neck, shoulders, elbows and wrists and the right hip. For the first year improvement had followed massage and medical treatment. For the past four and one-half years the right hip had gradually become stiff and painful in walking. When first seen by Dr. Myers in February, 1898, there was some spasm but no shortening. Motion of hip: Flexion 16 degrees, abduction 10 degrees, external rotation 10 degrees. A short traction hip-splint was at once applied and is still worn. There had been no pain since June, 1898, and the man considered himself greatly improved.

Dr. Ketch recalled the case of a man in whom the terrific pain of a sarcoma of the femur had not been relieved by powerful narcotics, but had been relieved for a time by traction made with a long hip-splint, and afterwards, as more convenient, with a short splint.

### FRACTURE OF NECK OF FEMUR IN AN INFANT.

Dr. Myers showed a specimen of fracture of the neck of the femur in a child eight months old. A large amount of callus was present within and without the periosteum. There was a lateral displacement of the lower fragment inward one-third the diameter of the bone. There was no change in the length of the bone. No history could be obtained except that the injury must have occurred before the fifth month.

### A NEW PELVIC REST.

Dr. Myers also showed a pelvic rest, especially well suited for the application of spica bandages, which included the trunk and thighs, as it could remain in place until the spica was fully applied, and could then be easily withdrawn. It was made of a piece of sheet steel, $\frac{1}{4}$x$1\frac{1}{2}$x14 inches, bent upon itself so as to form three sides of a square. The ends were hammered out so as to form oblong planes about three inches broad and five inches long. When in use one of the planes rested upon the table and the other supported the sacrum while the upright connecting them was directed towards the feet.

